# Successful treatment of dupilumab in Kimura disease independent of IgE: A case report with literature review

**DOI:** 10.3389/fimmu.2022.1084879

**Published:** 2022-12-14

**Authors:** Boyun Yang, Hanxiao Yu, Minyue Jia, Wo Yao, Ran Diao, Bohui Li, Yongfang Wang, Ting Li, Liuya Ge, Huiying Wang

**Affiliations:** ^1^ Department of Allergy, Second Affiliated Hospital of Zhejiang University School of Medicine, Hangzhou, Zhejiang, China; ^2^ Clinical Research Center, Second Affiliated Hospital of Zhejiang University School of Medicine, Hangzhou, Zhejiang, China; ^3^ Department of Ultrasound, Second Affiliated Hospital of Zhejiang University School of Medicine, Hangzhou, Zhejiang, China; ^4^ Outpatient Care Department, Second Affiliated Hospital of Zhejiang University School of Medicine, Hangzhou, Zhejiang, China

**Keywords:** Kimura disease, anti-IgE, dupilimab, anti-IL-4/IL-13, biologics, treatment

## Abstract

Kimura disease (KD) is a rare and benign chronic inflammatory disease of unknown cause. It is characterized by subcutaneous granuloma of soft tissues in the head and neck region, increased eosinophil count, and elevated serum IgE. Currently, no definitive treatments are recommended. A 57-year-old Chinese man was diagnosed with KD after 7 years of slow subcutaneous masses growth. The patient underwent treatment of oral glucocorticoids for 1 year, but the masses recurred as the dosage was tapered down. Subsequent anti-IgE therapy of omalizumab administered subcutaneously at 450 mg/day at a 4-week interval did not show improvement. The size of masses and serum IgE and circulating eosinophils did not decrease significantly after 19 cycles of continuous treatment. Ultimately, switched strategy of dupilumab was applied at an initial dose of 600 mg, followed by 300 mg every 2 weeks for 4 months. This treatment demonstrated dramatical effects with reduced masses in each area and fast dropdown of eosinophil counts, while the high level of serum IgE remained without changes. Recently, different biologics including anti-IgE, anti-IL-5, and anti-IL-4/IL-13 have been applied to treat KD with satisfied results and help to explore the pathogenesis of this rare disease. To our knowledge, this is the first report that demonstrates the effects of two different biologics in the same patient and reveals the impressive clinical efficacy of dupilumab to treat KD independent of IgE. Therefore, further investigation of the underlying mechanism and the development of diagnosis and treatment of KD is valuable.

## 1 Introduction

Kimura disease (KD) is a rare chronic inflammatory disease of unknown etiology (1). It manifests as slowly enlarging, painless subcutaneous masses, commonly affecting the subcutaneous soft tissues and lymph nodes of the head and neck, especially the salivary glands, parotid glands, and lymph nodes around (2). In particular, it is often accompanied by the infiltration of eosinophils and mast cells in subcutaneous tissues, elevated serum IgE, and circulating eosinophils. In addition, nephrotic syndrome occurs in up to 60% of the KD patients (3). Most of the reported cases come from Asian countries, including China, Japan, and India, generally occurring during the second to fourth decades of life, with a male-to-female ratio between 3.5:1 and 6:1 (4).

The etiology and pathogenesis of KD remain unclear. Recent studies suggest that it might be associated with allergic reactions, viruses or parasites interfering with the immune system, and even with arthropod bites (5). It is a benign disorder but prone to relapse. Conventional therapeutical strategies for KD include surgical excision, oral glucocorticoids, and radiation, but the optimal choice remains controversial. The broad application of biologics in clinics brings new resolution for this disease. Herein, we report a case of KD undergoing the treatment of omalizumab and dupilumab sequentially and analyzed the underlying mechanisms of different efficacy.

## 2 Case presentation

**Figure 1:**
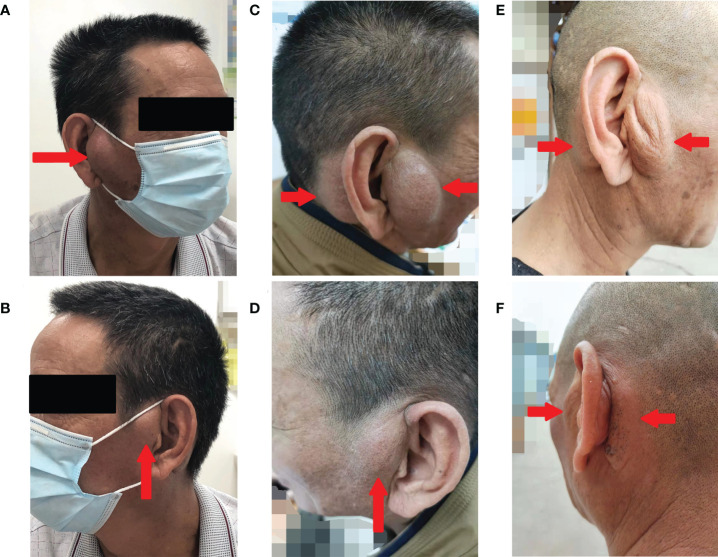
Photographs of the bilateral anterior and posterior ears of the patient. Before the treatment **(A, B)**, after 19 months of treatment with omalizumab **(C, D)**, and after 16 weeks of treatment with dupilumab **(E, F)** (arrows indicate tumorous regions).

**Figure 2:**
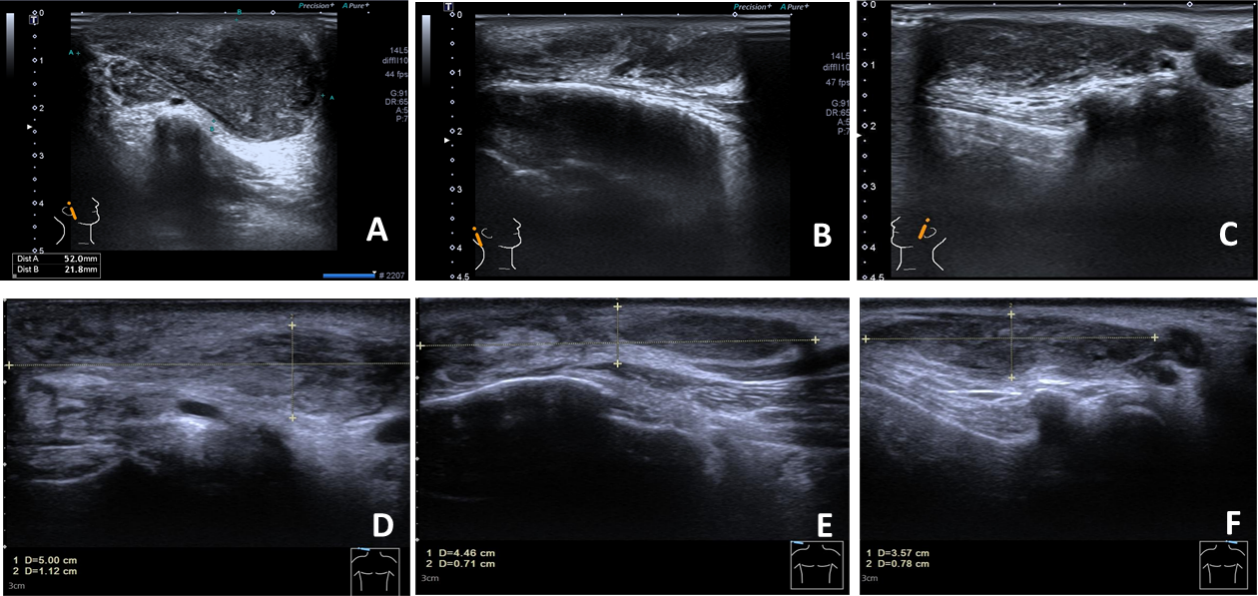
The anterior and posterior ears’ color Doppler ultrasound. The right anterior mass is in the size of 5.2×2.2 cm and has a heterogeneous hypoechoic–isoechoic appearance (right ear in front, right ear behind, and left ear in front); **(A–C)** before treatment; **(D–F)** after 16 weeks of treatment with dupilumab.

**Figure 3:**
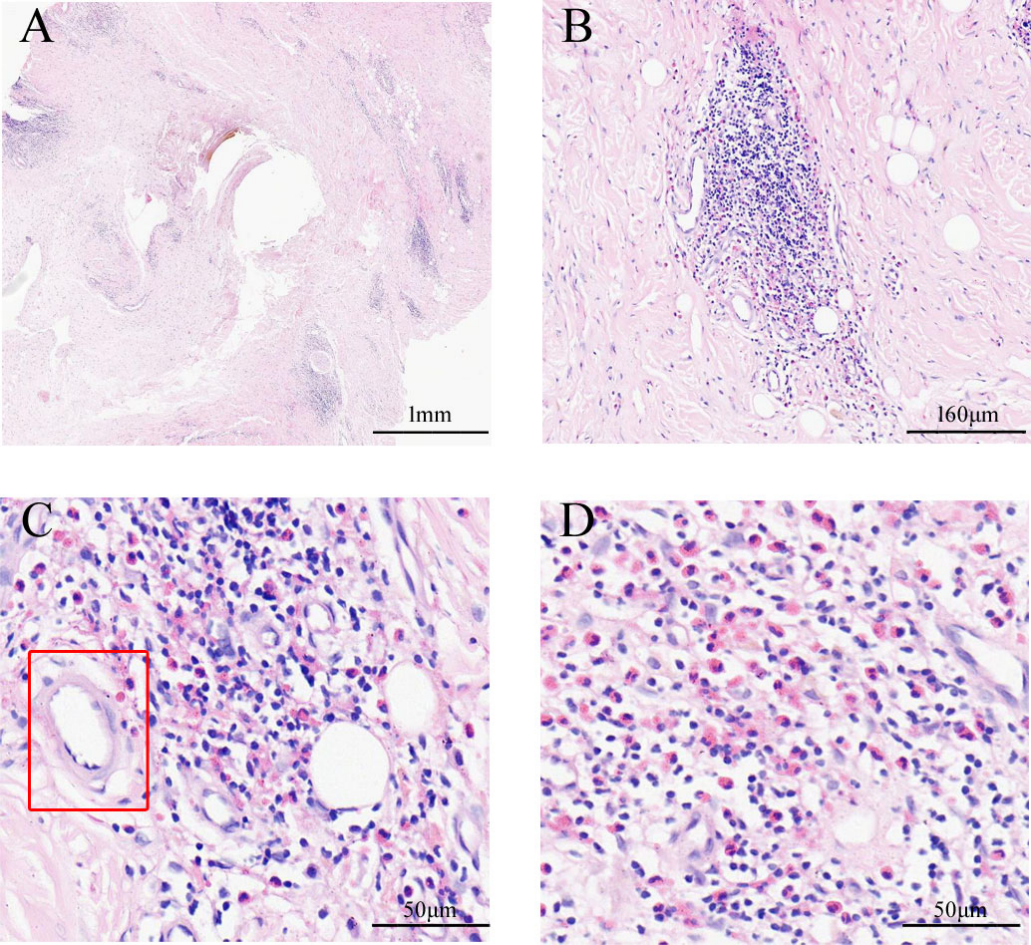
The pathological characteristics of ear masses. The gross view of pathological sections **(A)**. In the cortical region, lymphoid tissue hyperplasia with numerous eosinophils infiltrating **(B)**; hyperplasia of postcapillary venules with hyalinosis **(C, D)**; the marked region with the rectangle in C shows hyaline blood vessels (Hematoxylin and Eosin staining).

A 57-year-old Chinese man complained of bilateral anterior and posterior auricular painless, progressively enlarging masses for more than 7 years, accompanied with slight itching of the skin. No pain was felt on palpation. Physical examination showed anterior and posterior masses around both ears, the right anterior being the most obvious (**Figures 1A, B**). Lymph nodes nearby were not touched. Routine blood tests showed the increased number of eosinophils as 1.64 × 10^9^/L (22.8% of the total white blood cells), with decreased hemoglobin level at 113 g/L (normal, 131–172 g/L). Serum immunology tests demonstrated a dramatically high level of serum IgE over 2,000 IU/ml (normal range is <100 IU/ml), together with high immunoglobulin M (IgM) at 2.39 g/L (normal, 0.4–2.3 g/L). The counting number of reticulocyte erythrocytes, sedimentation rate (ESR), and urinary renal function were normal. Bone marrow biopsy suggested low-degree local hyperplasia than normal and a slight increase in acidophils. Ultrasound examination showed low echo masses in the front and back of the bilateral ear, patchy low echo area in the left parotid gland, and multiple lymph nodes enlargement in and around the bilateral parotid gland (**Figures 2A–C**). Histological sections of the mass tissue behind both ears revealed a large amount of eosinophils infiltration in the fibrous tissue and vascular hyperplasia with hyaline degeneration (**Figure 3**). The diagnosis of KD was proved based on his clinical manifestations, increased peripheral blood eosinophils counting, elevated serum IgE, and pathological evidence. He was treated with corticosteroids (prednisone) in the Department of Hematology for 1 year, initial at 20 mg/day for 8 months, then tapering down to 5 mg/day. The mass responded well under treatment of prednisone, while it recurred during the tapering of the medicine. Then, he underwent anti-IgE therapy and withdrew the prednisone.

**Figure 4:**
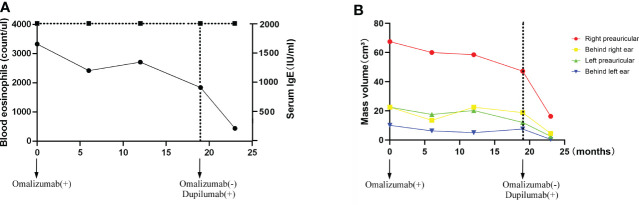
Time-course changes in the levels of eosinophil count and serum IgE **(A)**; the change in mass volume in each region during treatment **(B)**.

Omalizumab was administered to the patient subcutaneously at 450 mg/day at a 4-week interval. After four cycles of omalizumab, Chinese traditional medicine was added for adjuvant treatment. Yet, no obvious improvements have been found after long-term therapy of 19 cycles; the masses did not shrink, and circulating eosinophils did not go down significantly (**Figures 1C, D**, **4**). Serum IgE was measured four times at several time points as before omalizumab treatment and 6, 12, and 19 months after omalizumab treatment, with each time higher above 2,000 IU/ml (upper detection limit). We seem to have reached an impasse.

With the patient’s consent, switched strategy of dupilumab was applied at an initial dose of 600 mg, followed by 300 mg every 2 weeks for 4 months. The bilateral masses began to shrink since the second week and were more pronounced by the end of the first treatment course (16 weeks) (**Figures 1E, F**, **4B**). Ultrasound examination also showed clear reduction in the size of the anterior and posterior ear masses upon completion of the therapy compared with the size before (**Figures 2D–F**). No adverse reactions to dupilumab were complained of. Although the IgE level in the serum was still >2000 IU/ml, the eosinophil count returned to normal after the dupilumab therapy (**Figure 4A**). The treatment regimen and follow-up are still ongoing. This study of treatment of KD using the antibodies omalizumab and dupilumab was approved by the Ethics Committee of the Second Affiliated Hospital of Zhejiang University School of Medicine (Approval No. 20220608), and prior written informed consent was obtained from the patient.

## 3 Discussion

In the present report, we described a case of KD with successful treatment of dupilumab after failure of anti-IgE therapy. KD was first described as “eosinophilic proliferative lymphogranuloma” by Kimm and Szeto in China in 1937 along with the detailed pathological features described by KM et al. in Japan in 1948 (6). The pathogenesis of KD remains unclear, and there is no definitive treatment for it. Multiple therapeutic approaches have been performed to the clinical work in the past. Surgical excision is the mainstay of therapy; also oral cortisol, cytotoxic therapy, and radiation have been used (7). A clinical analysis reveals that the most successful treatments include a combination of surgery and radiation therapy (8). The developments of new techniques provide more simple and effective methods; for example, photodynamic therapy is a good regional debulking therapy (9).

Deeper knowledge of the immunological pathogenesis and appearance of new biologics greatly help in the treatment of KD. A Th2 cell immune response from the very beginning was suggested due to its impressive characteristics of elevated serum IgE, peripheral blood eosinophilia, and infiltration of eosinophils in tissues. Recent findings of its correlation to IgG4-related diseases also enhance this viewpoint (10–12). Ohta et al. found that KD patients have higher Th2 cells in the peripheral blood and elevated expression of IL-5, RANTES, and eotaxin in the lesions (13). Chen from China found that the expression of IL-21 and phosphorylated extracellular signal regulated kinase 1/2 (pERK1/2) in KD patients was correlated with its recurrence, which suggested that pERK1/2 pathway might be considered as a potential prognostic indicator in KD (14). Nonaka et al. found that the lesion of KD patients expressed increased levels of IL-4 (15). Recently, Kiyoshima et al. showed direct evidence with the existence of CD4+ GATA3+ T cells in the affected lymph node from patients with KD (16). These CD4+ GATA3+ T cells play a crucial role in IgE production *via* IL-4 and IL-5. These studies provide more targets for the therapy of KD.

Anti-IgE therapy has been tried first with some efficacy. In 2015, Nonaka et al. reported the first trial of a study of anti-IgE (omalizumab) treatment to three Japanese patients with KD uncontrolled by surgery and achieved successful results with decreased size of tumorous region and peripheral blood basophil and eosinophils’ counts (17). Anti-IL-5 mAb seems a promising regimen for KD, since it could inhibit the production, viability, recruitment, and lifespan of eosinophils in the blood and tissues. However, as Kinoshita reported, Mepolizumab (anti-IL-5 mAb) reduced the size and density of the mass in the upper limb together with the pathological changes in reduced eosinophils in the tissue instead of fibrosis (18). Recently, Szeto et al. demonstrated a successful treatment of benralizumab with the long-standing efficacy of clinical improvement (19). Dupilumab also has been applied by Hsuan-Yu Huang et al. and Yuichi Teraki et al. with complete relief after 8–10 month of therapy (20, 21).

In this case, the patient first tried anti-IgE therapy with omalizumab after relapse of the masses. Different from a previous report by Nonaka, it seems ineffective without any changes in our patient, even if the treatment period lasts to 19 cycles. One possible reason might be the insufficient dosage, since the serum IgE is more than 2,000 IU/ml. Another possibility is that IgE is not the key role of the mechanism. Inspite the elevation of serum IgE level, knowledge on the real role it plays in Kimura’s disease is lacking. Previous reports on the therapy of cyclosporin A and corticosteroidin showed a decrease in eosinophil level and persistent high serum IgE level (16, 22, 23). Thus, failure of anti-IgE therapy might happen in real clinical work.

On the contrary, dupilumab demonstrated a quick and dramatic efficacy in this patient with shrank mass and decreased eosinophils. Without the decrease in serum IgE level, the size of masses was remarkably smaller. This is in consistence with Nonaka’s finding that the high expression of IL-4 in the lesions and the inhibition of IL-4 by dupilumab could reverse the proliferation. According to the known clinical investigations, Th2 cytokines such as IL-4 and IL-5 are the major factors in the pathogenesis of KD. The eosinophilic aggregation and IgE synthesis are usually through IL-4 and IL-5 (24). The inhibition of IL-4 and IL-5 can effectively reduce the eosinophils in the lesion side and independent of IgE. This persistent high level of serum IgE unassociated with the improvement of tumor size is a strong support to our postulation that the successful treatment of dupilumab is independent of IgE.

To our knowledge, this is the first report that demonstrates the effects of two different biologics in the same patient and reveals the impressive clinical efficacy of dupilumab to treat KD independent of IgE. Therefore, it is valuable to further investigate the underlying mechanism and the development of diagnosis and treatment of KD.

## 4 Conclusion

In summary, we presented a typical KD patient, which was comprehensively confirmed by combining clinical manifestations, serological testing, and pathological biopsy. Biological therapy was applied after relapse with oral glucocorticoids. As a rare disease, the treatment for KD is still underexplored with very poor experience. Our case actually adds new evidence of anti-IgE and anti-IL-4/IL-13 therapy in KD.

## Data availability statement

The original contributions presented in the study are included in the article/supplementary material. Further inquiries can be directed to the corresponding author.

## Ethics statement

The studies involving human participants were reviewed and approved by the Ethics Committee of the second affiliated hospital of Zhejiang university school of medicine (Approval No. 20220608). The patients/participants provided their written informed consent to participate in this study. Written informed consent was obtained from the individual(s) for the publication of any potentially identifiable images or data included in this article.

## Author contributions

HYW was the guarantor and revised the manuscript. BYY and HXY wrote the manuscript and collected the data. MYJ interpreted the ultrasound pictures and provided professional comments. WY, RD, BHL, YFW, TL and LYG attended the treatment and follow-up of the patients. All authors contributed to the article and approved the submitted version.
